# Primary Biliary Neuroendocrine Tumors: A Systematic Review of Surgical Management, Oncologic Outcomes and Implications for Personalized Care

**DOI:** 10.3390/jpm16090445

**Published:** 2026-08-24

**Authors:** Anna Paspala, Dimitrios K. Vlachos, Dionysios Prevezanos, Panagiotis Dorovinis, Nikolaos Machairas, Stylianos Kykalos, Evangelos Tagkalos, Georgios C. Sotiropoulos

**Affiliations:** 1 Department of Liver Transplantation & Hepatobiliary Surgery, National and Kapodistrian University of Athens, 11527 Athens, Greece; dvlachos10@gmail.com (D.K.V.); prevedio@hotmail.com (D.P.); pdorovinis@gmail.com (P.D.); vtagalos@gmail.com (E.T.); gsotirop@yahoo.com (G.C.S.); 2 2nd Department of Surgery, National and Kapodistrian University of Athens, 11527 Athens, Greece; nmachair@gmail.com (N.M.); kykalos@gmail.com (S.K.)

**Keywords:** biliary neuroendocrine tumors, cholangiocarcinoma, extrahepatic bile duct, neuroendocrine neoplasms, systematic review, biliary tract tumors, personalized medicine

## Abstract

**Background/Objectives**: Primary biliary neuroendocrine tumors (PBilNETs) are exceptionally rare biliary tract neoplasms that are frequently misdiagnosed preoperatively as cholangiocarcinoma because of overlapping clinical and radiological findings. This systematic review aimed to summarize overall evidence regarding presentation, diagnostic evaluation, surgical management, and outcomes of PBilNETs. **Methods**: A systematic search of PubMed, Scopus, and Embase databases was performed according to PRISMA guidelines for studies published between January 2000 and December 2025. Studies including adult patients with histologically confirmed and surgically treated PBilNETs were eligible. Data regarding demographics, symptoms, imaging findings, surgical treatment, histopathology, immunohistochemistry, and outcomes were extracted and analyzed. **Results**: Fifty-eight studies involving 79 patients met the inclusion criteria. Median age at diagnosis was 49 years, with female predominance. Obstructive jaundice, abdominal pain, and pruritus were the most common presenting symptoms. Most tumors originated from the hilar or extrahepatic bile ducts. Preoperative diagnosis was challenging, as most lesions were initially considered cholangiocarcinomas. Surgical resection was the main therapeutic approach and included bile duct excision with biliary reconstruction, pancreaticoduodenectomy, or hepatic resection according to tumor location. Histopathological analysis demonstrated predominantly well- or moderately differentiated neuroendocrine neoplasms with frequent chromogranin A and synaptophysin positivity. Favorable long-term outcomes were reported, with high postoperative survival and limited recurrence during follow-up. **Conclusions**: PBilNETs remain diagnostically challenging because of their rarity and nonspecific presentation; however, they appear to exhibit a less aggressive biological behavior than conventional biliary adenocarcinomas. Surgical resection remains the cornerstone of treatment, while further multicenter studies are required to optimize diagnostic and therapeutic strategies. The findings also support an individualized multidisciplinary approach integrating clinical presentation, advanced imaging, histopathological grading, and immunohistochemical profiling to optimize personalized management of these rare tumors.

## 1. Introduction

Neoplasms of the biliary tract are relatively uncommon, with cholangiocarcinoma (CCA) representing the predominant histological subtype and accounting for the vast majority of malignancies arising in this anatomical region [1,2]. In contrast, neuroendocrine tumors (NETs) of the biliary tract are exceptionally rare, accounting for approximately 0.2–2% of all gastroenteropancreatic neuroendocrine neoplasms (GEP-NENs) and less than 1% of primary biliary tract malignancies [2,3]. Among biliary NETs, lesions arising from the extrahepatic bile ducts are reported more frequently than hilar and intrahepatic tumors, although precise epidemiological estimates remain difficult because of the limited number of published cases. Primary biliary neuroendocrine tumors (PBilNETs) are therefore among the rarest neuroendocrine neoplasms, with current knowledge derived almost exclusively from isolated case reports and small case series [3,4].

The pathogenesis of PBilNETs remains poorly understood. Neuroendocrine cells are sparsely distributed within the biliary epithelium, which may partially explain the low incidence of these tumors [2,5]. Several hypotheses have been proposed, including the development of neuroendocrine differentiation in the setting of chronic inflammation and intestinal metaplasia, as well as origin from multipotent epithelial progenitor cells capable of divergent differentiation [6]. Despite these theories, the molecular mechanisms underlying tumorigenesis in PBilNETs remain poorly characterized.

From a clinical perspective, PBilNETs most commonly present with symptoms related to biliary obstruction, including jaundice, pruritus, and abdominal discomfort, reflecting their anatomical location within the biliary tree [7,8]. Functional tumors with hormone-related symptoms are distinctly uncommon, and most lesions are non-secreting [9]. Radiological findings are typically nonspecific, frequently demonstrating biliary strictures or mass-forming lesions that are indistinguishable from CCA, which represents the principal differential diagnosis [2,10].

Preoperative diagnosis of PBilNETs remains particularly challenging as its clinical presentation closely mimics that of other more common tumors such as CCA [11]. Consequently, in the majority of reported cases, the diagnosis is established only after surgical resection through histopathological and immunohistochemical evaluation [2,11,12]. As previously mentioned, this diagnostic uncertainty frequently leads to management strategies that mirror those applied for CCA. For patients with resectable PBilNETs, complete surgical resection remains the treatment of choice and is associated with favorable oncologic outcomes [10,11]. Compared with conventional biliary adenocarcinomas, these tumors tend to exhibit a more indolent clinical course and more favorable prognosis, although outcomes vary according to tumor grade and stage. Nevertheless, the rarity of PBilNETs and the heterogeneity of the available data limit the ability to establish standardized diagnostic and therapeutic algorithms [10].

Although several systematic reviews have previously summarized the available literature on biliary neuroendocrine tumors, the evidence base has expanded in recent years with the publication of additional cases and contemporary pathological classifications. Furthermore, previous reviews were primarily descriptive and predated several important developments in the diagnosis, classification, and management of neuroendocrine neoplasms. Moreover, previous reviews were largely descriptive and did not specifically focus on surgically treated, histologically confirmed PBilNETs. An updated systematic synthesis is therefore warranted to provide a contemporary overview of their clinicopathological characteristics, diagnostic challenges, surgical management, and oncologic outcomes. Accordingly, the aim of the present study was to systematically review the available evidence on surgically treated PBilNETs, comprehensively characterize their clinicopathological features and management strategies, and identify knowledge gaps relevant to future research. Given the rarity and biological heterogeneity of these tumors, individualized diagnostic evaluation and multidisciplinary treatment planning remain essential. By integrating the currently available evidence, this review seeks to support personalized clinical decision-making while providing a foundation for future advances in molecular characterization, functional imaging, and precision-based management of PBilNETs.

## 2. Materials and Methods

### 2.1. Study Design and Search Strategy

A systematic review of the literature was conducted in accordance with the Preferred Reporting Items for Systematic Reviews and Meta-Analyses (PRISMA) guidelines (Table S1). The review protocol was prospectively registered in the Open Science Framework (OSF) and is publicly available (DOI: 10.17605/OSF.IO/ATKMF). Two investigators (A.P. and D.K.V.) independently performed the literature search, screened titles and abstracts, assessed full-text articles for eligibility, extracted the study data, and evaluated the methodological quality of the included studies. Any disagreements at each stage of the review process were resolved through discussion until consensus was reached. The search was restricted to studies published from January 2000 onwards to provide a contemporary cohort of patients managed according to modern diagnostic, pathological, and surgical standards. Furthermore, only adult patients (≥18 years) were included to minimize clinical heterogeneity related to pediatric disease.

**Figure 1:**
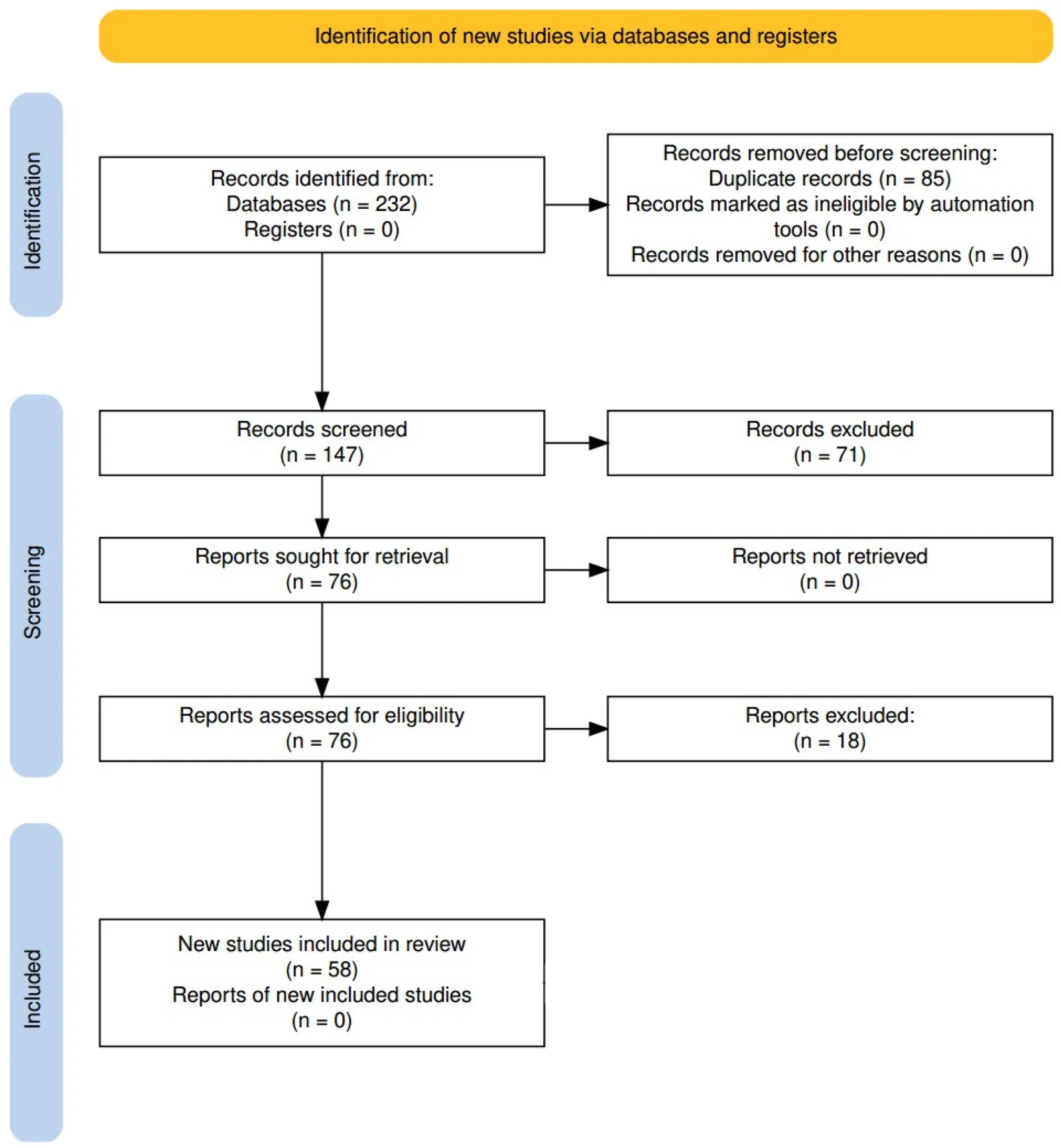
PRISMA flow diagram of the included studies.

The search strategy combined Medical Subject Headings (MeSH) and free-text terms related to primary biliary neuroendocrine tumors, including “biliary carcinoid”, “biliary neuroendocrine tumor”, “biliary neuroendocrine neoplasm”, “biliary NET”, “primary biliary NET”, “extrahepatic bile duct neuroendocrine tumor”, and related synonyms using the Boolean operators AND and OR. The complete electronic search strategy for each database is provided in Supplementary Table S2. Only articles published in English were considered for inclusion. The PRISMA flow diagram summarizes the study selection process (Figure 1).

### 2.2. Eligibility Criteria

Studies were considered eligible if they met the following inclusion criteria: (1) case reports or case series; (2) adult patients aged ≥18 years; (3) primary neuroendocrine tumors arising from the intrahepatic or extrahepatic biliary tree; (4) histopathological confirmation of diagnosis by biopsy or surgical specimen; and (5) resectable disease at presentation. The restriction to adult patients was predefined to ensure a clinically homogeneous study population, as pediatric PBilNETs are exceedingly rare and differ from adult cases regarding clinical presentation, biological behavior, therapeutic strategies, and long-term outcomes.

Exclusion criteria were as follows: (1) review articles, editorials, or conference abstracts; (2) pediatric cases; (3) gallbladder NETs; (4) unresectable or metastatic disease at presentation; (5) poorly differentiated neuroendocrine carcinomas; (6) functioning (hormone-secreting) neuroendocrine tumors; (7) mixed neuroendocrine–non-neuroendocrine neoplasms, including mixed adenoneuroendocrine carcinoma (MANEC) or mixed neuroendocrine–non-neuroendocrine neoplasms (MiNEN); and (8) studies with insufficient or non-extractable data. Functioning NETs, poorly differentiated neuroendocrine carcinomas (NECs), mixed neuroendocrine–non-neuroendocrine neoplasms (MiNENs), and patients with unresectable or metastatic disease were excluded to create a more homogeneous study population focused on surgically treated, well-differentiated primary biliary NETs. These entities differ substantially in biological behavior, diagnostic pathways, therapeutic management, and prognosis, and their inclusion would have introduced considerable clinical heterogeneity, limiting meaningful interpretation of surgical and oncologic outcomes.

### 2.3. Study Selection

All records identified through the database search were imported into a reference management system, and duplicate entries were removed. Titles and abstracts were independently screened to identify potentially eligible studies. Full-text articles were subsequently reviewed for inclusion based on the predefined eligibility criteria. Titles, abstracts, and subsequently full-text articles were independently screened by two reviewers (A.P. and D.K.V.) according to the predefined eligibility criteria. Disagreements regarding study eligibility were resolved through discussion until consensus was achieved.

### 2.4. Data Extraction

Data were extracted using a standardized data collection form. The following variables were recorded: patient demographics (age, sex), tumor characteristics (location within the biliary tree, size, histological grade), clinical presentation, diagnostic modalities, type of surgical intervention, pathological findings, and available follow-up outcomes. Only studies providing sufficient clinical and pathological data were included in the final analysis.

### 2.5. Data Synthesis

Given the rarity of PBilNETs and the heterogeneity of the included studies, a qualitative synthesis of the data was performed. Descriptive statistics were used to summarize clinicopathological characteristics and treatment outcomes.

### 2.6. Quality Assessment

The methodological quality of the included studies was independently assessed using the Joanna Briggs Institute (JBI) Critical Appraisal Checklists by two reviewers (A.P. and D.K.V.). The JBI Checklist for Case Reports was applied to individual case reports, while the JBI Checklist for Case Series was used for studies reporting multiple patients. Each study was evaluated across the relevant domains according to the JBI guidance, with each criterion rated as Yes, No, Unclear, or Not Applicable. Disagreements were resolved through discussion and consensus. Given that the available evidence consisted exclusively of case reports and small case series, the quality assessment was performed to evaluate the completeness and transparency of reporting rather than to exclude studies from the review.

## 3. Results

### 3.1. Included Studies

A total of 58 studies (52 case reports and 6 case series), which comprised 79 patients who underwent surgical treatment for PBilNETs were included in our systematic review [5,6,13,14,15,16,17,18,19,20,21,22,23,24,25,26,27,28,29,30,31,32,33,34,35,36,37,38,39,40,41,42,43,44,45,46,47,48,49,50,51,52,53,54,55,56,57,58,59,60,61,62,63,64,65,66,67,68].

### 3.2. Main Outcomes

Table 1 highlights the main outcomes of the current study. The median age of included patients with PBilNETs was 49 years, whereas the male to female ratio was 0.68 (32 male and 47 female). The majority of patients (67/79, 87.3%) were symptomatic at presentation. Regarding clinical presentation, obstructive jaundice was the most common symptom (48/79, 60.8%), followed by abdominal pain (35/79, 44.3%) and pruritus (19/79, 24.1%). A combination of these symptoms was reported in 36 patients (45.6%), whereas only 10 patients (12.7%) were asymptomatic at the time of diagnosis. Preoperative CA 19-9 levels were reported in 40 patients, of whom 11 (27.5%) had elevated values. Preoperative imaging studies were described in 64 (81%) patients. Specifically, among them abdominal ultrasound was used in 26 (40.6%) cases, Computed tomography (CT) in 47 (73.4%), Magnetic Resonance Imaging/Magnetic Resonance Cholangiopancreatography (MRI/MRCP) in 42 (65.5%), while Endoscopic Retrograde Cholangiopancreatography (ERCP) and Endoscopic Ultrasound (EUS) in 32 (50%) and 16 (25%) patients, respectively. PET scanning as a preoperative imaging modality reported in 18 cases, whilst among them 6 patients had 68Ga-DOTATATE PET/CT scan. These imaging modalities were not mutually exclusive, as most patients underwent a multimodal diagnostic work-up incorporating two or more imaging techniques according to the clinical scenario. Therefore, the reported percentages reflect the frequency of use of each imaging modality rather than independent diagnostic pathways. Moreover, preoperative biopsy or brushing was described in 26 cases, while 17 had biliary stent preoperatively for jaundice.

With regard to the exact location of primary PBilNETs, as reported for 64 patients, 32 (50%) of included cases were diagnosed with a hilar lesion, 30 (46.9%) with extrahepatic lesions involving the common hepatic or common bile ducts and 2 (3.1%) patients were diagnosed with intrahepatic PBilNETs. Among the 54 patients in whom a preoperative diagnosis was reported, the most frequent diagnosis was Klatskin tumor (44.4%) followed by extrahepatic adenocarcinoma (16.6%) and only 12 (22.2%) patients had a preoperative diagnosis of PBilNETs.

### 3.3. Surgical Outcomes

All 79 patients underwent surgical resection; 52 (65.8%) underwent open extrahepatic biliary tree (BT) resection & Roux en Y reconstruction, 9 underwent pancreaticoduodenectomy (Whipple’s procedure) (12.1%) and 3 (4%) underwent pylorus-preserving pancreaticoduodenectomy. Nine cases (11.3%) had extended hepatectomy plus extrahepatic BT excision & Roux en Y reconstruction, while two (2.7%) cases underwent laparoscopic cholecystectomy and 2 (2.7%) orthotopic liver transplantation. Robotic left hepatectomy plus cholecystectomy was performed in one case, while one patient underwent laparoscopic extrahepatic biliary tree excision. Radical lymphadenectomy was reported in 58 patients (73.4%).

Concerning pathological findings, grade of PBilNETs was described in 58.2% (n = 46/79) of included patients, while Ki-67 index was mentioned in 33 (41.7%) cases. More precisely, among them 17 (36.9%) patients diagnosed with Grade 1 PBilNETs, while 19 (41.3%) and 10 (21.7%) with Grade 2 and Grade 3 PBilNETs, respectively. Lymph node metastasis was detected in 14 patients. Immunohistochemical analysis revealed positive expression of Chromogranin A (CgA) and Synaptophysin (SynA) in 94% and 73% of patients, respectively, whereas CD56 positivity was reported in 22.6% of cases.

### 3.4. Long-Term Outcomes

Forty-one studies (52%) reported long-term outcomes for 51 patients. Four (7.8%) patients received adjuvant chemotherapy, and two patients (3.9%) received adjuvant irradiation, postoperatively. During a median overall follow-up of 19 months (range = 1–240 months), 88% of patients were alive. Three (5.8%) patients reportedly presented with recurrence of disease and specifically with liver metastases. One patient underwent 3 hepatectomies after the first surgery due to recurrent liver metastases [47]. One patient died 5 months postoperatively due to disease progression.

### 3.5. Quality Assessment of the Included Studies

Methodological quality was assessed in 58 studies, comprising 48 case reports and 10 case series (S1). Case reports fulfilled a median of 8 of 8 JBI criteria (range 6–8), whereas case series fulfilled a median of 8 of 10 criteria (range 6–10). All case reports adequately described patient demographics, clinical history, current presentation, diagnostic assessment, intervention, and the principal clinical lesson. Post-intervention clinical condition was clearly reported in 30 of 48 reports (62.5%), while adverse or unanticipated events were explicitly addressed in 39 of 48 reports (81.2%). Among case series, diagnostic methods, demographic and clinical characteristics, and study-site information were consistently reported; the most frequent limitations concerned explicit consecutive and complete inclusion of participants and the limited use of statistical analysis in small descriptive series. No study was excluded on the basis of methodological quality.

## 4. Discussion

This systematic review represents one of the most extensive evaluations of primary biliary neuroendocrine tumors (PBilNETs) reported to date, including 79 surgically managed patients across 58 studies. Several clinically meaningful patterns emerge from this analysis. PBilNETs appear to primarily affect individuals in mid-adulthood and demonstrate a predominance among female patients, while their clinical presentation is largely dictated by biliary obstruction, most frequently manifesting as jaundice. A key observation is the ongoing difficulty in establishing a correct preoperative diagnosis, as most cases are initially considered to represent CCA, with only a minority accurately identified prior to surgery. In addition, both imaging modalities and preoperative tissue sampling exhibit suboptimal diagnostic performance. Despite these limitations, surgical treatment is associated with favorable outcomes, including high survival rates and a low incidence of recurrence, reflecting the relatively indolent nature of these tumors.

Accurate preoperative identification of PBilNETs remains particularly challenging. In the present series, only 22% of patients had a correct preoperative diagnosis of PBilNET, with the majority being initially misclassified as CCA or other biliary malignancies. As supported by prior reports, imaging findings are generally nonspecific and most often demonstrate biliary strictures or mass-like lesions that closely resemble CCA [2,10,28]. The frequent use of multiple complementary imaging modalities underscores the diagnostic complexity of PBilNETs. Despite comprehensive multimodal imaging, preoperative differentiation from cholangiocarcinoma remained challenging, with only 21.8% of patients receiving the correct diagnosis before surgery. These findings suggest that current imaging techniques should be regarded as complementary rather than individually diagnostic in the evaluation of suspected PBilNETs. Although computed tomography and magnetic resonance imaging are integral components of the diagnostic pathway, their ability to discriminate PBilNETs from other biliary malignancies is limited. Functional imaging techniques, particularly somatostatin receptor-based PET/CT (e.g., ^68Ga-DOTATATE or ^68Ga-DOTATOC PET/CT), have demonstrated excellent diagnostic performance in gastroenteropancreatic neuroendocrine neoplasms and may provide additional value when PBilNET is suspected. More recently, fibroblast activation protein inhibitor (FAPI) PET/CT has emerged as a promising imaging modality for CCA because of its high uptake in desmoplastic tumors [69]. Therefore, in selected patients in whom the differential diagnosis includes both PBilNET and CCA, these imaging modalities may provide complementary biological information, with somatostatin receptor PET favoring neuroendocrine differentiation and FAPI PET demonstrating greater affinity for cholangiocarcinoma. Nevertheless, evidence specifically addressing PBilNETs remains extremely limited, and the role of these advanced imaging techniques should be further evaluated before routine implementation can be recommended [70]. As a result, definitive diagnosis is most commonly established following surgical resection through histopathological and immunohistochemical assessment.

Similarly, preoperative tissue diagnosis remains of limited value. In this review, biopsy or cytological sampling was performed in a relatively small proportion of patients and yielded inconclusive or non-diagnostic results in many cases, consistent with existing literature [2,68]. This may be explained by the predominantly subepithelial growth pattern of these tumors, combined with the technical limitations inherent to endoscopic sampling techniques. Consequently, the absence of diagnostic confirmation on biopsy does not exclude malignancy, and surgical exploration is frequently undertaken based on radiological suspicion. These findings emphasize a significant limitation in the current diagnostic approach and highlight the need for improved strategies, although meaningful progress is likely constrained by the rarity of PBilNETs.

Surgical resection remains the primary therapeutic modality and its extent is largely determined by tumor location [2,71]. In the present cohort, most patients underwent extrahepatic bile duct resection with subsequent biliary reconstruction, whereas pancreaticoduodenectomy or hepatic resection was performed for distal and hilar tumors, respectively. In routine clinical practice, the operative strategy often mirrors that applied for CCA, reflecting the challenges associated with accurate preoperative differentiation. Lymphadenectomy was frequently performed, although its prognostic relevance in PBilNETs remains uncertain due to limited available data. Nevertheless, the evidence suggests that complete tumor resection is associated with favorable long-term outcomes and should be pursued whenever feasible.

From a histopathological perspective, PBilNETs are predominantly well- to moderately differentiated neoplasms, as indicated by the high proportion of grade 1 and grade 2 tumors in this analysis. Immunohistochemical evaluation consistently confirms neuroendocrine differentiation, with expression of markers such as CgA and synaptophysin [11,72]. The Ki-67 proliferation index remains a key parameter for grading and prognostic assessment, although it is not uniformly reported across studies [11]. Compared with biliary adenocarcinoma, PBilNETs appear to exhibit a less aggressive clinical course, which is consistent with the favorable survival outcomes observed in this cohort [2,5]. Interestingly, approximately one-fifth of graded tumors in the present review were classified as Grade 3. Although this proportion appears higher than that generally reported for gastroenteropancreatic neuroendocrine tumors, this observation should be interpreted cautiously given the limited number of cases, incomplete reporting of tumor grade, and the potential for publication bias. Nevertheless, it raises the possibility that PBilNETs may exhibit a different histopathological spectrum from NETs arising at more common gastrointestinal sites, a finding that warrants further investigation in larger collaborative studies.

The role of adjuvant therapy in PBilNETs also remains ill-defined. Only a minority of patients in the present analysis received postoperative chemotherapy or radiotherapy, reflecting the absence of disease-specific treatment recommendations and the limited available evidence [73]. Current management is therefore largely extrapolated from guidelines for gastroenteropancreatic neuroendocrine neoplasms, in which routine adjuvant therapy following complete surgical resection is generally not recommended because of insufficient evidence, although it may be considered on an individualized basis for selected patients with high-grade tumors, advanced-stage disease, or other adverse pathological features according to multidisciplinary team discussion [74,75]. From a personalized medicine perspective, decisions regarding adjuvant treatment should integrate tumor grade, Ki-67 proliferation index, resection margin status, lymph node involvement, functional imaging findings, patient comorbidities, and, where available, molecular profiling to tailor postoperative management to the individual patient. However, these factors have not yet been systematically evaluated in PBilNETs because of the rarity of the disease, and treatment decisions should thus remain individualized within experienced multidisciplinary teams.

Several limitations should be considered when interpreting these findings. The available evidence is derived exclusively from case reports and small case series, introducing potential selection and publication bias and limiting the strength of the conclusions. While our eligibility criteria were intentionally selected to provide a more homogeneous evaluation of surgically managed PBilNETs, they inevitably limit the generalizability of the findings and may overestimate the favorable oncologic outcomes observed. In addition, incomplete reporting of key variables, including Ki-67 index, lymph node status, and long-term follow-up, restricts the ability to perform more detailed analyses. It should also be acknowledged that several earlier reports predated contemporary WHO classifications and described these tumors using the historical term “carcinoid.” Whenever possible, the included cases were interpreted according to current WHO terminology based on the available pathological descriptions. Nevertheless, incomplete histopathological reporting in older publications may have resulted in some degree of classification uncertainty. Importantly, the present review includes only patients who underwent surgical resection, thereby reflecting outcomes in a highly selected subgroup with resectable disease. Data regarding patients who do not undergo resection—either because of unresectable or metastatic disease, misdiagnosis, clinical deterioration, or palliative management—remain largely unavailable in the available published literature. Consequently, the favorable survival and low recurrence rates observed in this review are likely influenced by this inherent selection bias and should not be extrapolated to the entire spectrum of PBilNETs. This limitation may significantly skew the perceived prognosis and biological behavior of PBilNETs and renders our current understanding of the natural history of these tumors partly incomplete and potentially biased.

**Figure 2:**
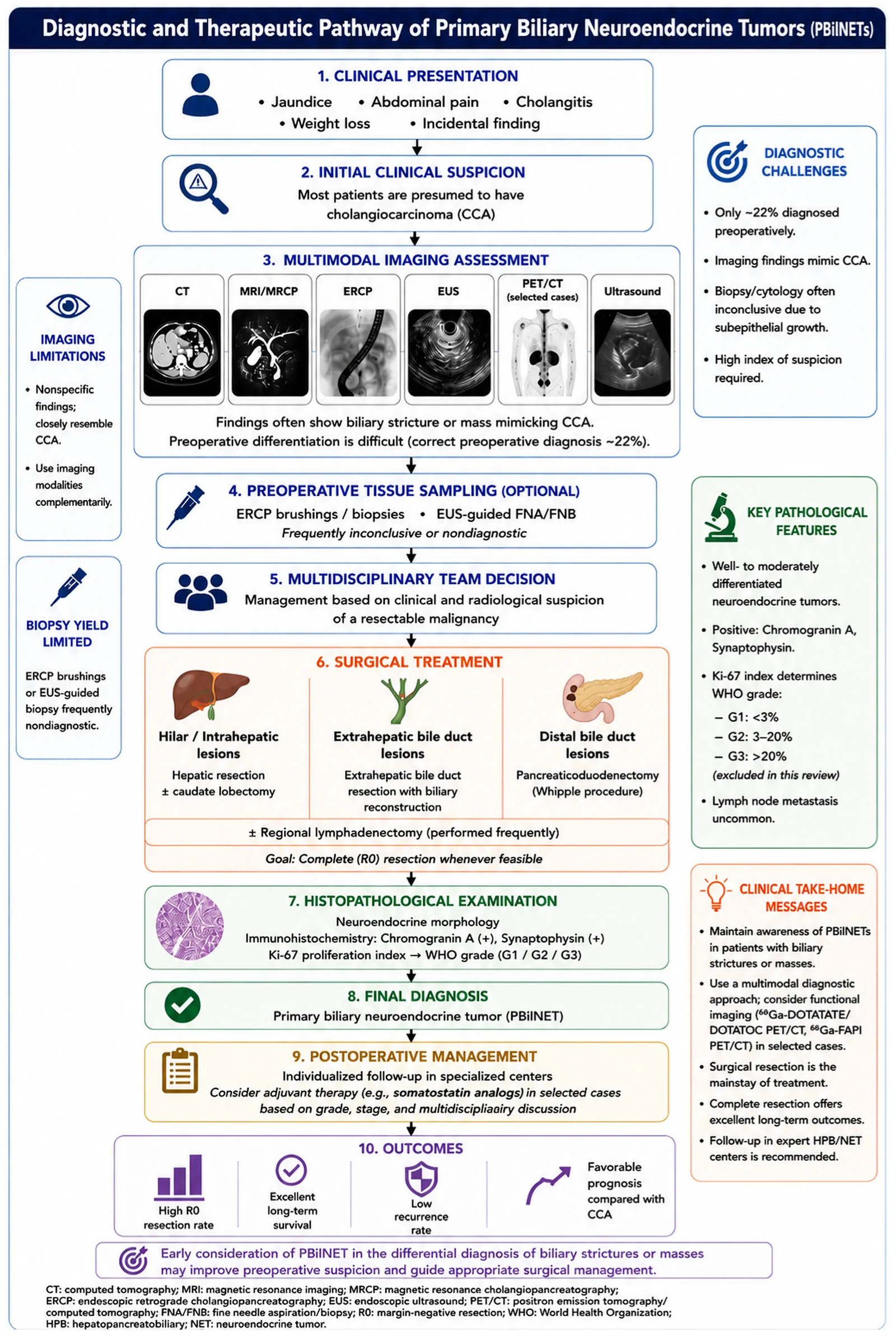
Personalized diagnostic and therapeutic pathway for patients with PBilNETs. Abbreviations: PBilNETs, primary biliary neuroendocrine tumors; CCA, cholangiocarcinoma; CT, computed tomography; MRI, magnetic resonance imaging; MRCP, magnetic resonance cholangiopancreatography; ERCP, endoscopic retrograde cholangiopancreatography; EUS, endoscopic ultrasound; PET/CT, positron emission tomography/computed tomography; ^68Ga-DOTATATE, gallium-68 DOTA-(Tyr3)-octreotate; ^68Ga-DOTATOC, gallium-68 DOTA-(Tyr3)-octreotide; FAPI, fibroblast activation protein inhibitor; FNA, fine-needle aspiration; FNB, fine-needle biopsy; WHO, World Health Organization; HPB, hepatopancreatobiliary; R0, microscopically margin-negative resection. This figure was generated with the assistance of ChatGPT (GPT-5.6 Luna, OpenAI, San Francisco, CA, USA) based on detailed instructions provided by the authors. The final content was reviewed and approved by all authors for scientific accuracy. Figure key: Arrows indicate the sequential diagnostic and therapeutic pathway. Icons represent the corresponding clinical, diagnostic, surgical, pathological, or outcome-related step. Box colors indicate the main thematic domains: blue, diagnostic assessment; orange, surgical management; green, histopathological diagnosis; purple, outcomes; gray, imaging modalities.

Given the exceptional rarity of PBilNETs, no dedicated evidence-based clinical guidelines currently exist to direct their diagnosis or management. Consequently, treatment strategies are generally extrapolated from broader recommendations for digestive neuroendocrine neoplasms and CCAs, despite the distinct biological and clinical characteristics of PBilNETs [75]. Current ENETS guidance emphasizes that management of rare neuroendocrine neoplasms should be individualized and undertaken within specialized multidisciplinary teams comprising hepatobiliary surgeons, gastroenterologists, medical oncologists, endocrinologists, radiologists, nuclear medicine physicians, and expert pathologists [76] (Figure 2). Such approach is particularly relevant for PBilNETs, in which accurate diagnosis, appropriate staging, and optimal treatment planning remain highly challenging. Indeed, discussion within dedicated neuroendocrine tumor multidisciplinary teams has been shown to substantially influence diagnosis and therapeutic decision-making, further supporting referral of these patients to expert centers whenever possible [77].

Future research should focus on the establishment of international multicenter registries in order to facilitate the systematic collection of clinicopathological and long-term outcome data for patients with PBilNETs. Given the rarity of these tumors and the potential for diagnostic misclassification, centralized pathological review and standardized reporting of histological grading and Ki-67 index may improve diagnostic accuracy and comparability across studies [78]. Furthermore, comprehensive genomic, transcriptomic, and molecular characterization of PBilNETs may improve biological understanding, refine prognostic stratification, and identify actionable therapeutic targets, thereby supporting future precision medicine strategies [79,80]. Functional imaging modalities, such as 68Ga-DOTATATE PET/CT, also warrant further evaluation regarding their role in preoperative diagnosis, staging, and postoperative surveillance. Finally, the role of systemic and neoadjuvant treatment strategies in advanced or borderline resectable disease remains poorly defined and ought to be explored in future collaborative studies.

## 5. Conclusions

Primary biliary neuroendocrine tumors constitute exceptionally uncommon neoplasms frequently mimicking CCA, hence rendering preoperative diagnosis highly challenging. Notwithstanding these diagnostic limitations, surgically treated PBilNETs appear to demonstrate favorable oncologic outcomes and a relatively indolent biological behavior compared with conventional CCA. Surgical resection remains the gold standard treatment and should be considered whenever feasible. Collectively, future collaborative studies integrating standardized pathological assessment, advanced imaging, and molecular profiling are needed to improve diagnostic accuracy and support personalized therapeutic strategies for these exceptionally rare tumors.

## Figures and Tables

**Table 1:** Clinicopathological characteristics and diagnostic findings.

Patient Characteristics	Number of Patients
Total number of patients	n = 79
Median age (range, in years)	49 (18–79)
Sex (female)	47/79 (59.4%)
Symptomatic patients	67/79 (87.3%)
Asymptomatic patients	10/79 (12.7%).
Combination of symptoms	36/79 (45.5%)
Elevated CA 19-9 (n = 40)	11/40 (27.5%)
Preoperative imaging studies (n = 64)	
US	26/64 (40.6%)
CT	47/64 (73.4%)
MRI/MRCP	42/64 (65.5%)
ERCP	32/64 (50%)
EUS	16/64 (25%)
68Ga-DOTATATE PET/CT	6/64 (9.3%)
Median tumor size (range, mm)	22 (2–80)
Preoperative biopsy/brushing	26
Preoperative biliary stenting	17
Tumor location (n = 64)	
Hilum	32/64 (50%)
Extrahepatic biliary tree	30/64 (46.9%)
Intrahepatic biliary tree	2/64 (3.1%)
Preoperative Diagnosis (n = 54)	
Klatskin	24/54 (44.4%)
Extrahepatic or pancreatic Adenocarcinoma	9/54 (16.6%)
NET	12/54 (22.2%)
Other	10/54 (18.5%)

Abbreviations: CA 19-9, Carbohydrate Antigen 19-9; US, Ultrasonography; CT, Computed Tomography; MRI, Magnetic Resonance Imaging; MRCP, Magnetic Resonance Cholangiopancreatography; ERCP, Endoscopic Retrograde Cholangiopancreatography; EUS, Endoscopic Ultrasound; PET, Positron Emission Tomography.

## Data Availability

The data underlying this article derive from those included in the relevant published articles.

## Supplementary Materials

The following supporting information can be downloaded at: https://www.mdpi.com/article/10.3390/jpm16090445/s1, Table S1: PRISMA_2020; Table S2: checklistThe complete electronic search strategy for each database.

## Abbreviations

The following abbreviations are used in this manuscript:

PBilNETs	Primary Biliary Neuroendocrine Tumors
CCA	Cholangiocarcinoma
NETs	Neuroendocrine Tumors
PRISMA	Preferred Reporting Items for Systematic Reviews and Meta-Analyses
MeSH	Medical Subject Headings
MANEC	Mixed AdenoNeuroendocrine Carcinoma
MiNEN	Mixed Neuroendocrine–Non-Neuroendocrine Neoplasm
CT	Computed Tomography
MRI	Magnetic Resonance Imaging
MRCP	Magnetic Resonance Cholangiopancreatography
ERCP	Endoscopic Retrograde Cholangiopancreatography
EUS	Endoscopic Ultrasound
PET	Positron Emission Tomography
CA 19-9	Carbohydrate Antigen 19-9
US	Ultrasonography
BT	Biliary Tree
CgA	Chromogranin A
SynA	Synaptophysin
