# Supplementary material for: Primary Biliary Neuroendocrine Tumors: A Systematic Review of Surgical Management, Oncologic Outcomes and Implications for Personalized Care
Source: J Pers Med. 2026 Aug 24;16(9):445. doi: 10.3390/jpm16090445 (PMC13608694; doi:10.3390/jpm16090445)
Supplement: Supplementary file 1 [file jpm-16-00445-s001.zip › jpm-4394038-The complete electronic search strategy for each database.pdf]

JBI Case reports											
**Study**	**Year**	**Q1 Demographics**	**Q2 History/timeline**	**Q3 Current clinical condition**	**Q4 Diagnostic tests/assessment**	**Q5 Intervention/treatment**	**Q6 Post-intervention condition**	**Q7 Adverse/unanticipated events**	**Q8 Take-away lessons**	**Yes count (/8)**	**Brief justification**
Nafidi et al.	2008	Yes	Yes	Yes	Yes	Yes	Yes	Yes	Yes	8	Core demographic, clinical, diagnostic, therapeutic and pathological details were reported. Follow-up and adverse events were clearly described.
Oshiro et al.	2016	Yes	Yes	Yes	Yes	Yes	Yes	Yes	Yes	8	Core demographic, clinical, diagnostic, therapeutic and pathological details were reported. Follow-up and adverse events were clearly described.
Park et al.	2014	Yes	Yes	Yes	Yes	Yes	Yes	Yes	Yes	8	Core demographic, clinical, diagnostic, therapeutic and pathological details were reported. Follow-up and adverse events were clearly described.
Sasatomi et al.	2013	Yes	Yes	Yes	Yes	Yes	Yes	Yes	Yes	8	Core demographic, clinical, diagnostic, therapeutic and pathological details were reported. Follow-up and adverse events were clearly described.
Terán et al.	2014	Yes	Yes	Yes	Yes	Yes	Unclear	Yes	Yes	7	Core demographic, clinical, diagnostic, therapeutic and pathological details were reported. Follow-up and/or explicit adverse-event reporting was limited or not clearly stated.
Zhang et al.	2018	Yes	Yes	Yes	Yes	Yes	Yes	Unclear	Yes	7	Core demographic, clinical, diagnostic, therapeutic and pathological details were reported. Follow-up and/or explicit adverse-event reporting was limited or not clearly stated.
Koo et al.	2019	Yes	Yes	Yes	Yes	Yes	Yes	Yes	Yes	8	Core demographic, clinical, diagnostic, therapeutic and pathological details were reported. Follow-up and adverse events were clearly described.

Caglikulekci et al.	2006	Yes	Yes	Yes	Yes	Yes	Yes	Yes	Yes	8	Core demographic, clinical, diagnostic, therapeutic and pathological details were reported. Follow-up and adverse events were clearly described.
Chaouch et al.	2019	Yes	Yes	Yes	Yes	Yes	Yes	Yes	Yes	8	Core demographic, clinical, diagnostic, therapeutic and pathological details were reported. Follow-up and adverse events were clearly described.
Han et al.	2023	Yes	Yes	Yes	Yes	Yes	Unclear	Unclear	Yes	6	Core demographic, clinical, diagnostic, therapeutic and pathological details were reported. Follow-up and/or explicit adverse-event reporting was limited or not clearly stated.
Hosonuma et al.	2007	Yes	Yes	Yes	Yes	Yes	Yes	Yes	Yes	8	Core demographic, clinical, diagnostic, therapeutic and pathological details were reported. Follow-up and adverse events were clearly described.
Jevdokimov et al.	2023	Yes	Yes	Yes	Yes	Yes	Yes	Yes	Yes	8	Core demographic, clinical, diagnostic, therapeutic and pathological details were reported. Follow-up and adverse events were clearly described.
Kiya et al.	2021	Yes	Yes	Yes	Yes	Yes	Yes	Yes	Yes	8	Core demographic, clinical, diagnostic, therapeutic and pathological details were reported. Follow-up and adverse events were clearly described.
Liu et al.	2018	Yes	Yes	Yes	Yes	Yes	Yes	Yes	Yes	8	Core demographic, clinical, diagnostic, therapeutic and pathological details were reported. Follow-up and adverse events were clearly described.
Nesi et al.	2006	Yes	Yes	Yes	Yes	Yes	Yes	Yes	Yes	8	Core demographic, clinical, diagnostic, therapeutic and pathological details were reported. Follow-up and adverse events were clearly described.
Safwan et al.	2015	Yes	Yes	Yes	Yes	Yes	Yes	Yes	Yes	8	Core demographic, clinical, diagnostic, therapeutic and pathological details were reported. Follow-up and adverse events were clearly described.

Saito et al.	2024	Yes	Yes	Yes	Yes	Yes	Yes	Yes	Yes	8	Core demographic, clinical, diagnostic, therapeutic and pathological details were reported. Follow-up and adverse events were clearly described.
Sánchez-Cabús et al.	2017	Yes	Yes	Yes	Yes	Yes	Unclear	Yes	Yes	7	Core demographic, clinical, diagnostic, therapeutic and pathological details were reported. Follow-up and/or explicit adverse-event reporting was limited or not clearly stated.
Sassi et al.	2020	Yes	Yes	Yes	Yes	Yes	Yes	Yes	Yes	8	Core demographic, clinical, diagnostic, therapeutic and pathological details were reported. Follow-up and adverse events were clearly described.
Schmitt et al.	2008	Yes	Yes	Yes	Yes	Yes	Unclear	Yes	Yes	7	Core demographic, clinical, diagnostic, therapeutic and pathological details were reported. Follow-up and/or explicit adverse-event reporting was limited or not clearly stated.
Umezaki et al.	2019	Yes	Yes	Yes	Yes	Yes	Yes	Yes	Yes	8	Core demographic, clinical, diagnostic, therapeutic and pathological details were reported. Follow-up and adverse events were clearly described.
Jethava et al.	2013	Yes	Yes	Yes	Yes	Yes	Unclear	Unclear	Yes	6	Core demographic, clinical, diagnostic, therapeutic and pathological details were reported. Follow-up and/or explicit adverse-event reporting was limited or not clearly stated.
Athanasopoulos et al.	2011	Yes	Yes	Yes	Yes	Yes	Yes	Yes	Yes	8	Core demographic, clinical, diagnostic, therapeutic and pathological details were reported. Follow-up and adverse events were clearly described.
Boeți et al.	2019	Yes	Yes	Yes	Yes	Yes	Yes	Yes	Yes	8	Core demographic, clinical, diagnostic, therapeutic and pathological details were reported. Follow-up and adverse events were clearly described.

Choi et al.	2019	Yes	Yes	Yes	Yes	Yes	Yes	Yes	Yes	8	Core demographic, clinical, diagnostic, therapeutic and pathological details were reported. Follow-up and adverse events were clearly described.
Costin et al.	2017	Yes	Yes	Yes	Yes	Yes	Unclear	Unclear	Yes	6	Core demographic, clinical, diagnostic, therapeutic and pathological details were reported. Follow-up and/or explicit adverse-event reporting was limited or not clearly stated.
Danila et al.	2021	Yes	Yes	Yes	Yes	Yes	Unclear	Yes	Yes	7	Core demographic, clinical, diagnostic, therapeutic and pathological details were reported. Follow-up and/or explicit adverse-event reporting was limited or not clearly stated.
De Luca et al.	2013	Yes	Yes	Yes	Yes	Yes	Unclear	Unclear	Yes	6	Core demographic, clinical, diagnostic, therapeutic and pathological details were reported. Follow-up and/or explicit adverse-event reporting was limited or not clearly stated.
Ferreira-Silva et al.	2023	Yes	Yes	Yes	Yes	Yes	Unclear	Yes	Yes	7	Core demographic, clinical, diagnostic, therapeutic and pathological details were reported. Follow-up and/or explicit adverse-event reporting was limited or not clearly stated.
Gusani et al.	2008	Yes	Yes	Yes	Yes	Yes	Yes	Yes	Yes	8	Core demographic, clinical, diagnostic, therapeutic and pathological details were reported. Follow-up and adverse events were clearly described.
Hoepfner et al.	2017	Yes	Yes	Yes	Yes	Yes	Unclear	Yes	Yes	7	Core demographic, clinical, diagnostic, therapeutic and pathological details were reported. Follow-up and/or explicit adverse-event reporting was limited or not clearly stated.

Hosoda et al.	2016	Yes	Yes	Yes	Yes	Yes	Unclear	Yes	Yes	7	Core demographic, clinical, diagnostic, therapeutic and pathological details were reported. Follow-up and/or explicit adverse-event reporting was limited or not clearly stated.
Bhalla et al.	2012	Yes	Yes	Yes	Yes	Yes	Unclear	Yes	Yes	7	Core demographic, clinical, diagnostic, therapeutic and pathological details were reported. Follow-up and/or explicit adverse-event reporting was limited or not clearly stated.
Cappell et al.	2011	Yes	Yes	Yes	Yes	Yes	Unclear	Unclear	Yes	6	Core demographic, clinical, diagnostic, therapeutic and pathological details were reported. Follow-up and/or explicit adverse-event reporting was limited or not clearly stated.
Juturi et al.	2000	Yes	Yes	Yes	Yes	Yes	Yes	Unclear	Yes	7	Core demographic, clinical, diagnostic, therapeutic and pathological details were reported. Follow-up and/or explicit adverse-event reporting was limited or not clearly stated.
Khan et al.	2017	Yes	Yes	Yes	Yes	Yes	Unclear	Yes	Yes	7	Core demographic, clinical, diagnostic, therapeutic and pathological details were reported. Follow-up and/or explicit adverse-event reporting was limited or not clearly stated.
Khuroo et al.	2014	Yes	Yes	Yes	Yes	Yes	Yes	Yes	Yes	8	Core demographic, clinical, diagnostic, therapeutic and pathological details were reported. Follow-up and adverse events were clearly described.
Menezes et al.	2004	Yes	Yes	Yes	Yes	Yes	Yes	Yes	Yes	8	Core demographic, clinical, diagnostic, therapeutic and pathological details were reported. Follow-up and adverse events were clearly described.
Miyashita et al.	2020	Yes	Yes	Yes	Yes	Yes	Yes	Yes	Yes	8	Core demographic, clinical, diagnostic, therapeutic and pathological details were reported. Follow-up and adverse events were clearly described.

Radu et al.	2021	Yes	Yes	Yes	Yes	Yes	Unclear	Yes	Yes	7	Core demographic, clinical, diagnostic, therapeutic and pathological details were reported. Follow-up and/or explicit adverse-event reporting was limited or not clearly stated.
Raspanti et al.	2016	Yes	Yes	Yes	Yes	Yes	Unclear	Unclear	Yes	6	Core demographic, clinical, diagnostic, therapeutic and pathological details were reported. Follow-up and/or explicit adverse-event reporting was limited or not clearly stated.
Rykała et al.	2014	Yes	Yes	Yes	Yes	Yes	Unclear	Unclear	Yes	6	Core demographic, clinical, diagnostic, therapeutic and pathological details were reported. Follow-up and/or explicit adverse-event reporting was limited or not clearly stated.
Stavridi et al.	2008	Yes	Yes	Yes	Yes	Yes	Yes	Yes	Yes	8	Core demographic, clinical, diagnostic, therapeutic and pathological details were reported. Follow-up and adverse events were clearly described.
Taj et al.	2023	Yes	Yes	Yes	Yes	Yes	Unclear	Yes	Yes	7	Core demographic, clinical, diagnostic, therapeutic and pathological details were reported. Follow-up and/or explicit adverse-event reporting was limited or not clearly stated.
Todoroki et al.	2007	Yes	Yes	Yes	Yes	Yes	Yes	Yes	Yes	8	Core demographic, clinical, diagnostic, therapeutic and pathological details were reported. Follow-up and adverse events were clearly described.
Turrión et al.	2002	Yes	Yes	Yes	Yes	Yes	Yes	Yes	Yes	8	Core demographic, clinical, diagnostic, therapeutic and pathological details were reported. Follow-up and adverse events were clearly described.
Abe et al.	2017	Yes	Yes	Yes	Yes	Yes	Yes	Yes	Yes	8	Core demographic, clinical, diagnostic, therapeutic and pathological details were reported. Follow-up and adverse events were clearly described.

Kim et al.	2006	Yes	Yes	Yes	Yes	Yes	Yes	Yes	Yes	8	Core demographic, clinical, diagnostic, therapeutic and pathological details were reported. Follow-up and adverse events were clearly described.

**JBI Case series**														
**Study**	**Year**	**Design**	**Q1 Clear inclusion criteria**	**Q2 Standard/reliable condition measurement**	**Q3 Valid identification methods**	**Q4 Consecutive inclusion**	**Q5 Complete inclusion**	**Q6 Clear demographics**	**Q7 Clear clinical information**	**Q8 Outcomes/follow-up reported**	**Q9 Site/demographic information**	**Q10 Appropriate statistical analysis**	**Yes count (/10)**	**Brief justification**
Lee et al.	2016	Multicenter retrospective study	Yes	Yes	Yes	Unclear	Unclear	Yes	Yes	Yes	Yes	Yes	8	Study population, diagnosis, demographics and clinical/pathological outcomes were described. The principal concerns were explicit consecutive/complete inclusion and, in small descriptive series, limited statistical analysis.
Ishida et al.	2019	Retrospective single-center series	Yes	Yes	Yes	Yes	Yes	Yes	Yes	Unclear	Yes	Yes	9	Study population, diagnosis, demographics and clinical/pathological outcomes were described. The principal concerns were explicit consecutive/complete inclusion and, in small descriptive series, limited statistical analysis.

Jun et al.	2006	Four-case radiologic series	Unclear	Yes	Yes	Unclear	Unclear	Yes	Yes	Yes	Yes	N/A	6	Study population, diagnosis, demographics and clinical/pathological outcomes were described. The principal concerns were explicit consecutive/complete inclusion and, in small descriptive series, limited statistical analysis.
Kim et al.	2011	Three-center retrospective series	Yes	Yes	Yes	Unclear	Yes	Yes	Yes	Yes	Yes	Yes	9	Study population, diagnosis, demographics and clinical/pathological outcomes were described. The principal concerns were explicit consecutive/complete inclusion and, in small descriptive series, limited statistical analysis.
Lee et al.	2011	Four-case series	Unclear	Yes	Yes	Unclear	Unclear	Yes	Yes	Yes	Yes	N/A	6	Study population, diagnosis, demographics and clinical/pathological outcomes were described. The principal concerns were explicit consecutive/complete inclusion and, in small descriptive series, limited statistical analysis.
Podnos et al.	2003	Two-case series	Unclear	Yes	Yes	Unclear	Unclear	Yes	Yes	Yes	Yes	N/A	6	Study population, diagnosis, demographics and clinical/pathological outcomes were described. The principal concerns were explicit consecutive/complete inclusion and, in small descriptive series, limited statistical analysis.
Ferrone et al.	2007	Three-patient institutional series	Unclear	Yes	Yes	Unclear	Unclear	Yes	Yes	Yes	Yes	N/A	6	Study population, diagnosis, demographics and clinical/pathological outcomes were described. The principal concerns were explicit consecutive/complete inclusion and, in small descriptive series, limited statistical analysis.

Hong et al.	2014	Retrospective radiologic series	Yes	Yes	Yes	Yes	Yes	Yes	Yes	Yes	Yes	Yes	10	Study population, diagnosis, demographics and clinical/pathological outcomes were described. The principal concerns were explicit consecutive/complete inclusion and, in small descriptive series, limited statistical analysis.
Chopde et al.	2024	Four-case single-center series	Yes	Yes	Yes	Yes	Yes	Yes	Yes	Yes	Yes	N/A	9	Study population, diagnosis, demographics and clinical/pathological outcomes were described. The principal concerns were explicit consecutive/complete inclusion and, in small descriptive series, limited statistical analysis.
Maitra et al.	2000	Seven-case pathologic series	Yes	Yes	Yes	Unclear	Unclear	Yes	Yes	Yes	Yes	Yes	8	Study population, diagnosis, demographics and clinical/pathological outcomes were described. The principal concerns were explicit consecutive/complete inclusion and, in small descriptive series, limited statistical analysis.
